# The Rhetoric of Disenchantment: Ghost Belief and Secular Critique in Early Twentieth‐Century China

**DOI:** 10.1111/cogs.70158

**Published:** 2026-01-19

**Authors:** Ze Hong, Yuqi Chen

**Affiliations:** ^1^ Department of Sociology University of Macau; ^2^ Centre for Quantitative History, Faculty of Business and Economics University of Hong Kong

**Keywords:** Religious cognition, China, Secularization, Ghosts, Argumentation, Rhetoric

## Abstract

This study presents the first large‐scale empirical analysis of how ghosts and spirits were debated during China's early twentieth‐century secular transformation. Using a novel dataset of over 2000 digitized texts—including newspapers, periodicals, and essays from 1890 to 1949—we combine close reading, AI‐assisted annotation, and statistical modeling to examine rhetorical strategies surrounding supernatural belief. We find a clear asymmetry: critics emphasized theoretical arguments (e.g., science, rationality, education), while defenders relied more on empirical or anecdotal evidence. These patterns reflect broader institutional and cognitive shifts, including the rise of science as a dominant epistemology and the increasing use of psychological explanations to pathologize belief. While reformist elites often cast ghost belief as superstition, we also identify agnostic, cautious, and reconciliatory positions. By situating these debates within the broader context of Chinese cultural modernization, the study sheds new light on how supernatural belief became a contested domain and offers fresh tools for studying the cultural evolution of religious cognition.

## 1 Introduction

Throughout history, religion—broadly defined—has played a central role in human societies, shaping our worldview and affecting our everyday lives in significant ways. However, religious claims have always had doubters and skeptics (Schellenberg, 2007; Thiessen & Wilkins‐Laflamme, 2017), and the religious landscape of the world has changed substantially over the past few centuries (Clarke, 2004). Following the emergence of scientific institutions and the rise of mechanistic philosophy, doubts and critiques of religious doctrines in post‐Enlightenment Europe became rampant among intellectual elites (Berman, 1981). The fundamental validity of religion's truth claims came under scrutiny, and the traditional roles of religion in explaining, predicting, and controlling worldly events gradually gave way to new functions centered on community building and personal spiritual growth (Bruce, 2011; Horton, 1993; Taylor, 2007).

While considerable scholarly attention has been devoted to understanding this intellectual transition in Europe (Carroll, 2011; Li, 2007; Madra, 2015; Wootton, 2016), we know far less about how secularizing repertoires were articulated and contested in non‐Western print cultures (Basalla, 1967). In the present study, we use China as a case study to examine religious discourse in public print culture at the turn of the twentieth century, as a window into the cognitive and institutional mechanisms by which secular and religious frames competed in public reasoning. Unlike many other societies, China never adopted a national religion with a fully developed theology. However, its ideological system incorporated a recognition of supernatural entities, including gods, deities, and spirits, which played a significant role in shaping religious and social life (Palmer, 2017). These supernatural beings formed the theoretical foundation of magico‐religious practices such as rainmaking and dream divination, which persisted throughout premodern China (Hong, 2022; Hong, Slingerland, & Henrich, 2024). Historically, the existence of these supernatural entities was occasionally cast into doubt by Confucian scholars (Li, 2021), yet visible critiques of certain religious doctrines expanded in the early twentieth century among Western‐educated reformers, journalists, and officials writing in urban newspapers and periodicals. During this period, Western‐educated Chinese elites actively attacked religious beliefs and practices, dismissing them as “superstitious” and incompatible with modernity (Yang, 2021).

A key aspect of this transformation was the changing attitude toward ghosts and spirits. Throughout Chinese history, beliefs in ghosts and spirits were widespread, deeply embedded in everyday life, and reflected in ancestor worship, funeral rites, and various protective rituals (Poo, 2022). Importantly, these supernatural beings were not merely figures of folklore but were perceived as real agents capable of influencing human affairs. However, as Western scientific thought gained traction among Chinese intellectuals, such beliefs were increasingly framed as irrational and vestiges of a bygone era (Goossaert & Palmer, 2011). This shift was not simply a passive byproduct of modernization but part of an active, elite‐led effort to redefine China's intellectual and cultural foundations.

This paper focuses on the historical transformation of public print discourse in early twentieth‐century China, analyzing how expressed sentiments and arguments toward ghosts and spirits evolved. Drawing on digitized newspapers, periodicals, and intellectual discourses from the late Qing (mid‐nineteenth century–1912) and the Republican (1912–1949) periods, we examined how ghosts and spirits were discussed, criticized, or defended in public discourse. While this print discourse primarily reflects the views of an educated, often urban, elite rather than the entire populace, it is the essential source for this analysis. These media served as the central arena where influential modernizers, intellectuals, and state‐builders sought to frame the public debate against “superstition,” disseminate a scientific worldview, and ultimately construct a new, rational foundation for the Chinese nation. Therefore, examining these sources provides direct insight into the strategies used to publicly contest and redefine the boundaries of belief during this pivotal era. In addition to an in‐depth exploratory analysis of this unique dataset, we conducted preregistered analyses to determine whether critiques of belief in ghosts were primarily theoretical, challenging their plausibility, or empirical, emphasizing their failure to produce tangible effects. From a cognitive science perspective, this distinction is not merely descriptive but theoretically important, as it maps onto different modes of human reasoning. Theoretical arguments, which appeal to metaphysical plausibility, conceptual coherence, or compatibility with scientific worldviews, engage reflective, abstract reasoning processes that draw on counterfactual thinking and the evaluation of coherence within broader explanatory systems (Evans, 2008; Mercier & Sperber, 2017). By contrast, empirical arguments, rooted in specific events, observations, or testimonies, activate cognitive mechanisms that privilege concrete and salient information, such as episodic memory, narrative processing, and the heuristic that personal or trusted‐witness experience is especially credible (Schank & Abelson, 2014; Tversky & Kahneman, 1974). These differences have consequences for belief resilience: empirical claims, while compelling, are more vulnerable to contradictory evidence or selective reporting, whereas theoretical arguments, embedded in broader ontological frameworks, may provide more durable scaffolding for either sustaining or rejecting belief (Hong et al., 2024). Such a cognitive characterization of arguments builds on a body of work examining the cultural evolution of “ineffective” technological practices such as divination, dream interpretation, and taboos (Hong, 2022, 2024; Hong et al., 2024; Hong & Henrich, 2021). This line of research suggests that as modern scientific epistemology rises, such practices are often challenged at a foundational level—by rejecting the worldview that makes them plausible—rather than through a simple accumulation of disconfirming empirical evidence. The rejection of the supernatural, in this view, often involves a clash of entire theoretical frameworks (Hong, 2025). As will be shown, skeptics in this period often targeted the conceptual foundations of ghost belief, undermining its plausibility within a modern scientific framework (e.g., modern science precludes the existence of such beings), while defenders relied heavily on experiential validation through anecdotes and testimony (e.g., personal ghost experiences), consistent with our hypotheses. This asymmetry reflects well‐documented cognitive tendencies in processing abstract versus experiential information and offers a lens into the interaction between psychological constraints and the sociohistorical dynamics of secularization.

Beyond its immediate historical focus, this study has broader implications for the comparative study of religion and belief change. By examining these discourses, we contribute to wider debates on secularization, religious skepticism, and the intellectual transformation of societies undergoing rapid modernization. The findings illuminate how traditional supernatural beliefs are challenged, modified, or abandoned, offering insight into the cognitive and cultural mechanisms that shape shifts in religious thought. In doing so, this work engages with and extends secularization theory, which has long examined the declining social authority of religion in contexts of scientific and institutional modernity (Gorski & Altınordu, 2008; Hadden, 1987; Wallace, 1966). It also advances the literature on cultural evolution by showing how belief systems are molded through the interplay of cognitive biases and changing informational, institutional, and social environments (Henrich, 2016).

## 2 Method and dataset description

To systematically obtain intellectual discourse on ghosts/spirits in late Qing and early Republic China, we performed a keyword search for “guishen 鬼神” (ghosts and spirits) across several major digital archives: Late Qing Full‐text Database, Chinese Periodical Full‐text Database, and Newspapers in the online version of Shanghai Library (https://www.cnbksy.com/) and the Hantang Modern Newspapers Database (https://www.neohytung.com/). Together, these sources cover more than 10 million documents across approximately 20,000 periodicals published between 1833 and 1949.

Using an “exact match” search strategy and excluding metaphorical uses of *guishen* unrelated to supernatural beings (see Supporting Information for details), we initially retrieved 4382 texts. From these, 2222 were identified as relevant for analysis. To meaningfully annotate the extracted texts—many of which had not yet been OCR‐processed—we recruited research assistants proficient in Classical Chinese and trained them using a detailed annotation protocol. In parallel, we employed ChatGPT‐4o for machine‐assisted annotation. The model was guided by a detailed, structured prompt, the full text of which is available in our OSF repository (prompt.txt). This prompt provided the model with clear definitions for each coding category—for instance, explaining how to differentiate between abstract, theoretical discussions of *guishen* and specific, anecdotal accounts of supernatural encounters. To “train” the model for our specific task, the prompt included several illustrative examples for each category, a technique known as in‐context learning (Dong et al., 2024). This process does not alter the underlying model but conditions its output for the specific analytical task by providing a clear framework and examples within the query itself.

To ensure the consistency and reliability of this dual‐coding process, we conducted inter‐coder reliability checks on a subset of texts coded by all parties (both human and AI). We calculated Fleiss’ kappa, a robust statistical measure that assesses the level of agreement between multiple raters, correcting for the possibility that agreement occurred by chance. The resulting scores, which ranged from 0.3 to 0.6, indicate a fair to moderate level of agreement (see Supporting Information for methodology).

Each text was annotated for a number of features, with the present paper focusing on the following (full annotation guidelines and coding rubrics are available in the Supporting Information): (1) Whether the text affirms the existence of ghosts/spirits; (2) Whether belief in ghosts/spirits is portrayed as practically useful; (3) Whether empirical evidence is presented for or against their existence; (4) Whether theoretical arguments are offered for or against their existence; and (5) Whether belief in ghosts/spirits is discussed as having a psychological basis.

This annotation framework allowed us to systematically capture both the evaluative stance and argumentative structure of public discourse on ghosts and spirits during a critical period of intellectual transformation in China. By combining human and machine‐assisted coding, we aimed to balance interpretive nuance with consistency and scalability. It is worth noting that the annotation process posed significant challenges. Many texts were available only as image scans without OCR, making character recognition difficult. Moreover, articles from this era often blended Classical and early Vernacular Chinese, introducing semantic ambiguities that complicated consistent interpretation. Despite these difficulties, the annotated dataset represents a rare and valuable resource for the systematic study of public discourse in early twentieth‐century China. Importantly, beyond testing specific hypotheses, this dataset also enables rich descriptive insights—for example, tracking whether and how attitudes toward ghosts and spirits changed over time, and identifying recurring themes in public reasoning. In the following section, we present the results of our analysis, highlighting both descriptive patterns and the argumentative strategies through which belief in ghosts and spirits was affirmed, contested, or reinterpreted.

## 3 Results

### 3.1 Descriptive patterns and trends in ghost/spirit discourses

**Fig. 1:**
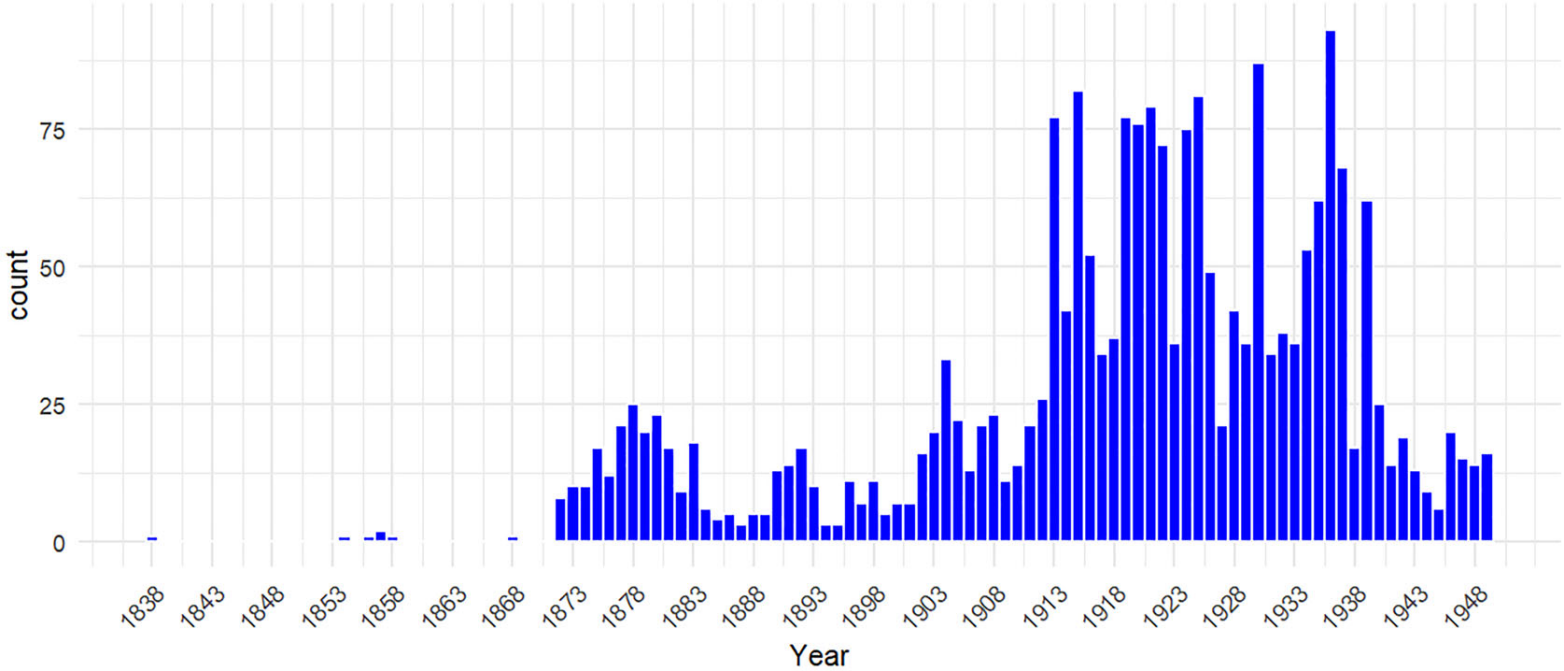
Temporal distribution of articles containing “ghosts/spirits.”

Fig. 1 shows the temporal distribution of articles mentioning ghosts/spirits between 1833 and 1949. As can be seen, most discussions regarding these supernatural entities revolve around the early Republican period (1910s−1930s). This trend partly reflects the surge in explicit debate over traditional beliefs during the New Culture Movement and the May Fourth Movement, when intellectuals actively questioned the foundations of Chinese culture, including its supernatural dimensions (Chow, 1960). It also reflects the dramatic expansion in the number of newspapers and periodicals following the establishment of the Republic of China, which facilitated the dissemination of modern and reformist ideas to a broader audience (MacKinnon, 1997). An analysis of the relative frequency of these terms is available in the Supporting Information.

**Fig. 2:**
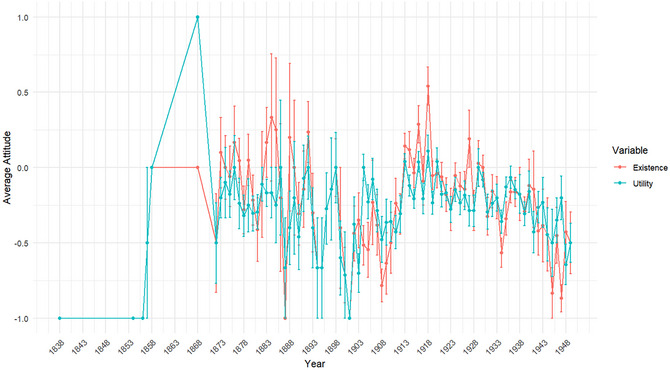
Temporal trends in attitudes toward the existence and utility of ghosts/spirits in Chinese periodicals (1833–1949). Error bars denote standard errors of the mean for each year. Both measures show substantial year‐to‐year variation.

Many of the texts express explicit opinions regarding the existence (71%; 1578 out of 2222) and utility (43%; 955 out of 2222) of ghosts and spirits. Fig. 2 presents the yearly average attitudes, where values of −1 and 1 represent negative and positive attitudes, respectively, and 0 indicates either vague or missing evaluations. As shown, although there is no clear temporal trend for either existence or utility, the average attitude in most years remains negative. Moreover, attitudes toward existence and utility are strongly correlated. A Pearson correlation test revealed a significant positive association between the two at the level of individual texts (*r* = .59, *p* < .001, 95% CI [0.56, 0.61]), suggesting that texts affirming the existence of ghosts and spirits were also more likely to portray them as practically useful.

The “utility” of ghosts and spirits is typically depicted in two ways. First, some texts describe belief in ghosts/spirits as useful because it brings tangible, material benefits, such as resolving illnesses, protecting families from harm, and attracting wealth or good fortune. Second, other texts emphasize the functional role of belief in supernatural entities, particularly its moral and psychological effects. As one text explains, even though the divine may be “remote and unknowable,” belief in spirits instills a sense of “reverence and fear,” prompting people to “avoid wrongdoing and refrain from evil.”^1^ This moralizing function reflects a long‐standing intellectual tradition that posits a positive relationship between religion and ethical behavior from classical arguments—such as the oft‐cited line traditionally attributed to Dostoyevsky, “if God doesn't exist, then everything is permitted”—to contemporary research that associates religious beliefs with morality (Norenzayan et al., 2014; Purzycki et al., 2016). In this view, religion serves as a vital mechanism for regulating human behavior in ways that reason or self‐interest alone cannot. However, during the Republican era, some voices began to question this fear‐based morality cultivated through religion. Critics argued that such morality was inferior to the kind fostered through education, which “instills a spirit of resilience and vitality”—qualities deemed essential to “break through all obstacles and truly reform today's society.”^2^

On the other hand, texts that emphasize the harm of belief in ghosts and spirits often label such beliefs explicitly as “superstition” (mixin 迷信)—a term that was positioned in stark opposition to science during the early Republican era (Huang, 2016). These critiques point to obsessive and irrational behaviors of believers, such as excessive spending on rituals, reliance on spirit mediums for everyday decisions, or neglect of medical treatment in favor of spiritual remedies. A telling example appears in a 1903 report from Shen Pao (a leading Shanghai‐based newspaper of the late Qing and early Republican periods known for its wide circulation and reformist orientation):

With excessive rains and unstable weather, the people have neglected proper care of their health, making them vulnerable to illness—especially children. In the western districts, the number of the sick has increased noticeably. As this comes at a critical time for agricultural work, the common folk are quietly anxious. There is talk of parading the plague gods to drive out the epidemic. Yet instead of promoting hygienic practices, they turn only to rituals and spirits—how foolish this is!^3^

Here, the author criticizes the worship of gods as a reaction to the ongoing epidemic as dangerously misguided, framing it as evidence of popular ignorance and misplaced faith. Such examples are numerous and show that debates over the utility of supernatural belief were not only philosophical but also deeply practical, especially in contexts of health, morality, and everyday decision‐making.

Nonetheless, a few texts depart from this dichotomy. Some authors criticize the *practical* efficacy of prayer to ghosts or spirits without explicitly denying their existence. For instance, a 1934 essay published in the *Hunan Provincial Government Gazette* takes aim at both blind believers and rigid skeptics. While acknowledging the possibility that ghosts and spirits may exist, the author insists that there is no compelling reason to believe they would intervene in human affairs. Drawing an analogy between divine supplication and bribing corrupt officials, the author writes:

Superstitious individuals often turn to divination and lots whenever they encounter problems they cannot solve on their own, placing their decisions in the hands of the gods…But are such gods really effective? Most of what the superstitious call “miraculous” is based on hearsay. Consider this from a commonsense perspective: Even a child asking something of a parent, or a friend asking something of a friend, is not guaranteed to get what they want. What connection do you have with ghosts and spirits? Why should they respond to your requests? And what do you offer in return? Are we to believe that a few kowtows, some incense, and flattering words are enough to win divine favor? If so, then ghosts and spirits would be no different from corrupt officials—accepting bribes like people in the mortal world. But such entities would be base and unworthy, and unfit to be considered true spirits. How could they possibly bring you blessings?^4^

While rhetorically sharp, the analogy is ironically undercut by the fact that bribing corrupt officials often does work (that is why they are corrupt!)—perhaps diminishing its persuasive force. In any case, the author's argument pivots to challenge the opposite extreme: those who categorically deny the existence of ghosts and spirits. He writes:

On the other hand, there are also people who categorically deny the existence of ghosts and spirits. They argue that, in this age of scientific enlightenment, everything real can be tested and verified by scientific methods. Since ghosts and spirits have no visible form, no audible sound, and cannot be empirically demonstrated, they must not exist. This view stands in direct opposition to the one we just discussed, and we cannot accept it either. Why? Because the principles of the universe are infinite, while human knowledge is limited. There are countless things that exist beyond the reach of current human understanding. If one's knowledge is limited, it is reasonable to suspend judgment. But to insist that what one cannot see or hear must not exist—this is short‐sighted. Ask yourself: Didn't people once claim that there was nothing in water, in air, or inside infectious diseases? Why did that change after Pasteur and others discovered microorganisms? Now everyone agrees that water and air contain microbes, and that infectious diseases involve harmful bacteria. By that logic, anything science cannot currently prove may simply lie beyond its present capabilities. We cannot say with certainty that ghosts and spirits do not exist.

This style of reasoning echoes certain strands of contemporary religious apologetics, which invoke the limits of science to defend the plausibility of supernatural or divine entities (Ferngren, 2022; Priest, 2023). In both historical and modern contexts, such arguments seek to preserve supernatural belief by framing it as an epistemically modest position in light of scientific uncertainty. Other texts go even further by emphasizing the unresolved nature of the question itself. One commentary, published in the reformist news outlet *New World*, for example, frames the existence of ghosts and spirits as an open scientific problem:

The question of whether ghosts and spirits exist is currently under investigation in countries around the world. We should neither assert their existence with certainty nor categorically deny it. Although China has long been known for its superstitious belief in ghosts and spirits, this stems from the ancient practice of using spiritual teachings to instill fear and restrain immoral behavior—serving as a supplement where the reach of law was insufficient. Later generations, failing to grasp this original intent, exaggerated such beliefs, bringing great ridicule upon our nation. This was certainly not the fault of the sages who first devised such teachings. Today, with the rise of science, belief in ghosts and spirits has gradually declined. However, in recent years, with the popularity of spiritualism, such beliefs have reemerged. Thus, the question of their existence has once again become the subject of intensive research. We should remain calm and patient, awaiting the final verdict from these researchers.^5^

Here, the author adopts a distinctly agnostic stance, rejecting both dogmatic belief and dogmatic disbelief, and casting the existence of ghosts and spirits as a question best left to future scientific inquiry.

**Fig. 3:**
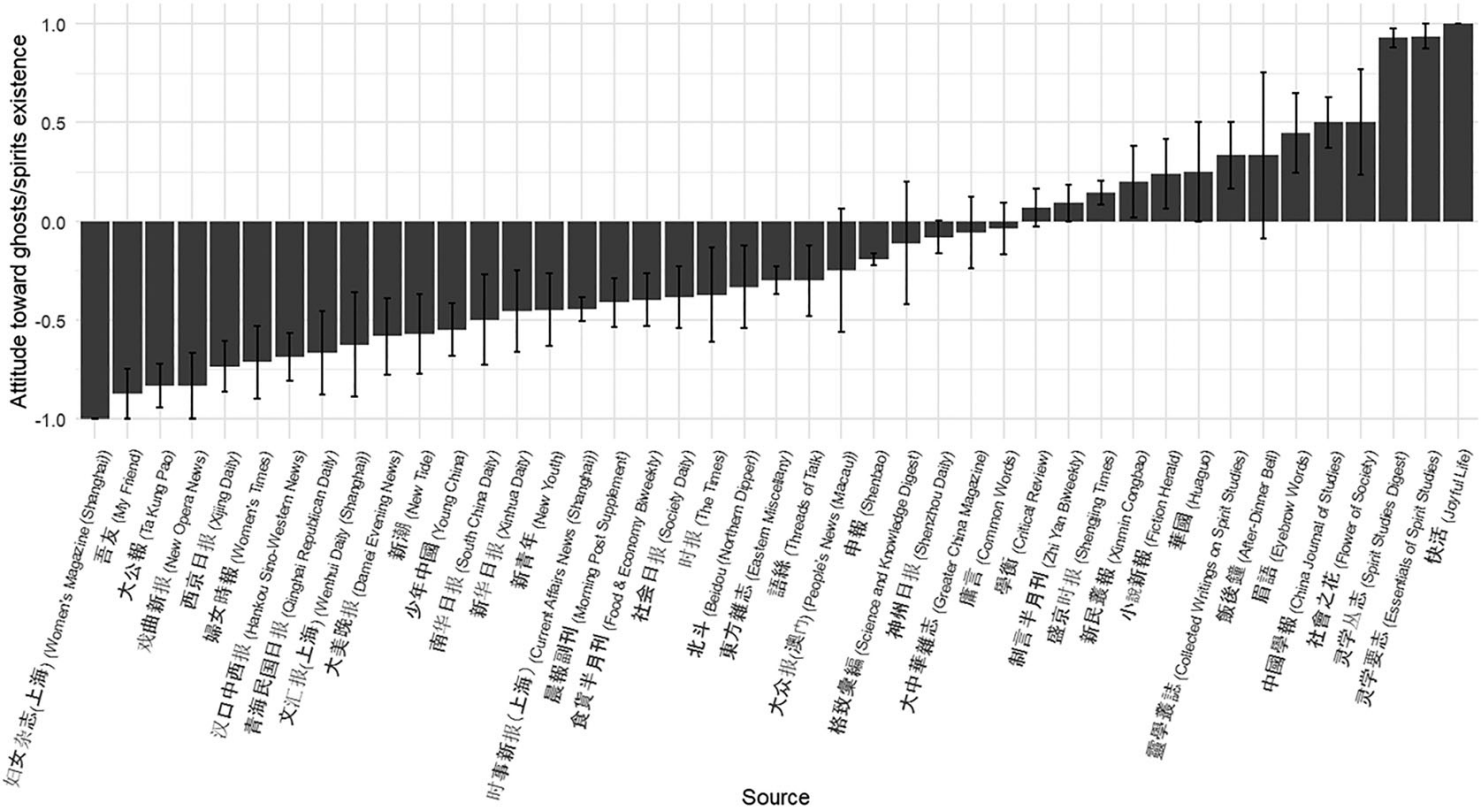
Average attitude toward the existence of ghosts/spirits by source (Chinese periodicals, 1833–1949). Error bars denote standard errors of the mean for each publication source.

Given the diversity of periodical and newspaper genres with different ideological stances, we further examined the average attitude toward ghosts and spirits by source. As shown in Fig. 3, different periodicals and newspapers varied widely in their treatment of these supernatural entities. Explicitly religious or spiritual outlets, such as *Spirit Studies Digest* and *Essentials of Spirit Studies*, displayed the most affirmative attitudes toward their existence. In contrast, progressive news outlets such as *Women's Magazine*, *My Friend*, and *Ta Kung Pao* overwhelmingly rejected the reality of ghosts and spirits. Notably, *Women's Magazine*—a publication committed to the physical and cultural emancipation of women—ran a special column in 1925 titled “*The Harm My Family Has Suffered from Believing in Ghosts and Spirits*,” which featured reader‐submitted accounts detailing the harmful consequences of excessive supernatural belief within their families, including unnecessary financial waste and the hypocrisy of religious and spiritual leaders.

### 3.2 Types of arguments for and against the existence of ghosts/spirits

In a series of prior studies, we have argued and demonstrated that criticisms of ghosts and spirits tend to be framed as rejections of their theoretical foundations, rather than as responses to empirical inadequacies (Hong, 2022, 2025; Hong et al., 2024; Hong & Henrich, 2021; Hong & Zinin, 2023). Here, we test a preregistered hypothesis^6^: namely, that most arguments *against* the existence of ghosts and spirits will take the form of theoretical critiques, whereas arguments *in favor* of their existence will more often rely on empirical justifications.

**Fig. 4:**
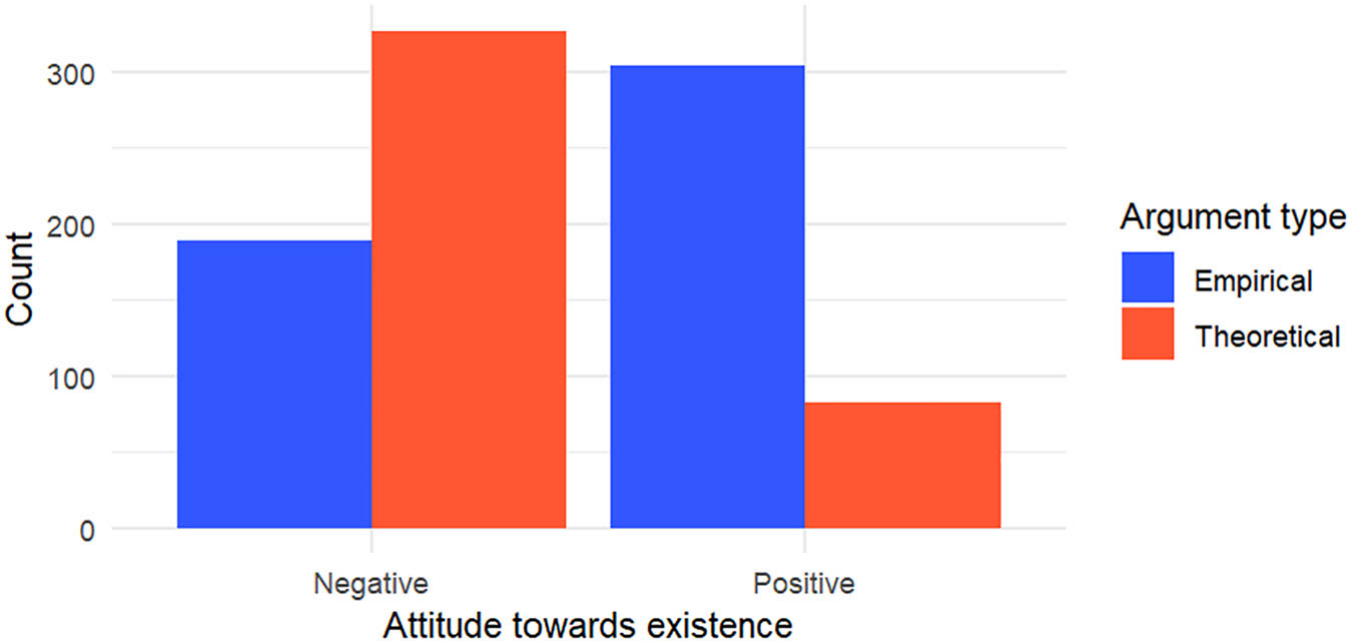
Counts of argument types by attitude toward the existence of ghosts/spirits.

To test this, we classified the relevant arguments in the extracted texts as either theoretical or empirical, following the criteria outlined in the Methods section. To give readers a clearer sense of how these argument types were expressed, we provide representative examples in Table 1. Specifically, “theoretical” arguments refer to those that challenge (or defend) the *plausibility* or *necessity* of supernatural entities from a conceptual or metaphysical standpoint. In contrast, “empirical” arguments involve appeals to specific events, observations, or individual experiences that are framed as evidence either for or against the existence of ghosts and spirits. We then compared the frequency of theoretical and empirical arguments across texts expressing either a positive or negative stance toward the existence of supernatural beings (see Fig. 4). As predicted, theoretical arguments were significantly more common in texts rejecting the existence of ghosts/spirits, whereas empirical arguments predominated in texts affirming their existence. This asymmetry supports the hypothesis that skeptics tend to rely on abstract or scientific reasoning to delegitimize supernatural belief, while believers are more likely to ground their claims in observed or narrated experiences.

**Table 1:** Examples of theoretical and empirical arguments for and against the existence of ghosts and spirits

Types of arguments	Theoretical	Empirical
Example (for)	“The Book of Changes says: ‘The essence and energy transform into things, wandering souls change their forms’. Therefore, one should understand the states of ghosts and spirits. Between heaven and earth, what is not a thing? What is not a soul? What is not a transformation?”^7^	Liu Bai, a native of Nanyi, was wild and unruly, staunchly disbelieving in ghosts—those who brought it up were often mocked … One night, while passing through a village, he heard footsteps behind him… turning back repeatedly, he saw nothing. Realizing something was wrong, he shouted, “If you're a real man, come out and face me!”… Suddenly, a palm appeared out of thin air and struck his face—so painful he fled in terror… The next morning, red marks dotted his cheeks like plum blossoms, lasting for years… From that day on, he never dared doubt the existence of spirits again.^8^
Example (against)	“I sigh at how our country's people believe in ghosts, and I laugh at how our ignorant countrymen fear ghosts without realizing that it is their own psychological and neurological processes at work—thus allowing superstition to persist for thousands of years without being dispelled.”^9^	“Despite my father's and my attempts to persuade her otherwise, she remains unshaken in her beliefs, even in the face of unexpected disasters. It's deeply lamentable that she busies herself with these rituals, especially the monthly Buddha worshipping sessions. Despite her diligent and sincere efforts, she gains no benefits; on the contrary, it results in a significant waste of resources.”^10^

Given that different publication venues may exhibit distinct attitudes toward ghosts and spirits, we performed a hierarchical logistic regression analysis to formally test the association between a text's stance on the existence of ghosts/spirits and its likelihood of including a theoretical justification, while accounting for variability at the publication source level. The outcome variable was whether the text employed a theoretical justification (coded as 1 if present, 0 if absent). The key predictor was the text's stance on existence (coded as 1 for rejection of ghosts/spirits, 0 for affirmation; note that this is different from how the variables are coded in the original dataset), with year of publication (centered) included as a covariate, and a random intercept for publication source to account for clustering.

Results show a strong and statistically significant relationship between rejecting belief in ghosts/spirits and the use of theoretical justifications. Specifically, texts that rejected the existence of ghosts or spirits are significantly more likely to invoke theoretical arguments (*β* = 1.53, *SE* = 0.18, *z* = 8.67, *p* < .001). This corresponds to an odds ratio of approximately 4.6, indicating that rejection of supernatural entities increases the odds of using a theoretical justification by over fourfold. Additionally, there is a modest but significant temporal trend: over time, the use of theoretical justifications declined slightly (*β* = −0.013, *SE* = 0.004, *z* = −2.89, *p* = .004) after adjusting for stance and source‐level variation, though we caution against overinterpreting this association given its exploratory nature.

Published discourses affirming the existence of ghosts and spirits were often empirical in nature. For example, *The China Times (Shanghai)* reported the following story:

In Mujiao, the proprietor of a cosmetics shop, Hu, died suddenly last year from a brief illness, leaving behind a wife and two sons—the elder aged sixteen, the younger aged five. A few days ago, the elder son also died of illness. The mother, deeply attached to her children, was so grief‐stricken that she no longer wished to live. Suddenly, Hu's spirit was said to have possessed the younger son and spoken: “Mother, do not grieve; this is your son's fate. While alive, the elder son committed many misdeeds and acted willfully. Now he has been judged by the underworld authorities and punished, cast into the realm of hungry ghosts, and is presently in the household of the [surname] family on [certain] Street. He will be reborn in Jiangbei.” The mother, skeptical, went in person to verify the claim. It was found that on that very day, that family had indeed welcomed the birth of a baby boy.^11^

**Fig. 5:**
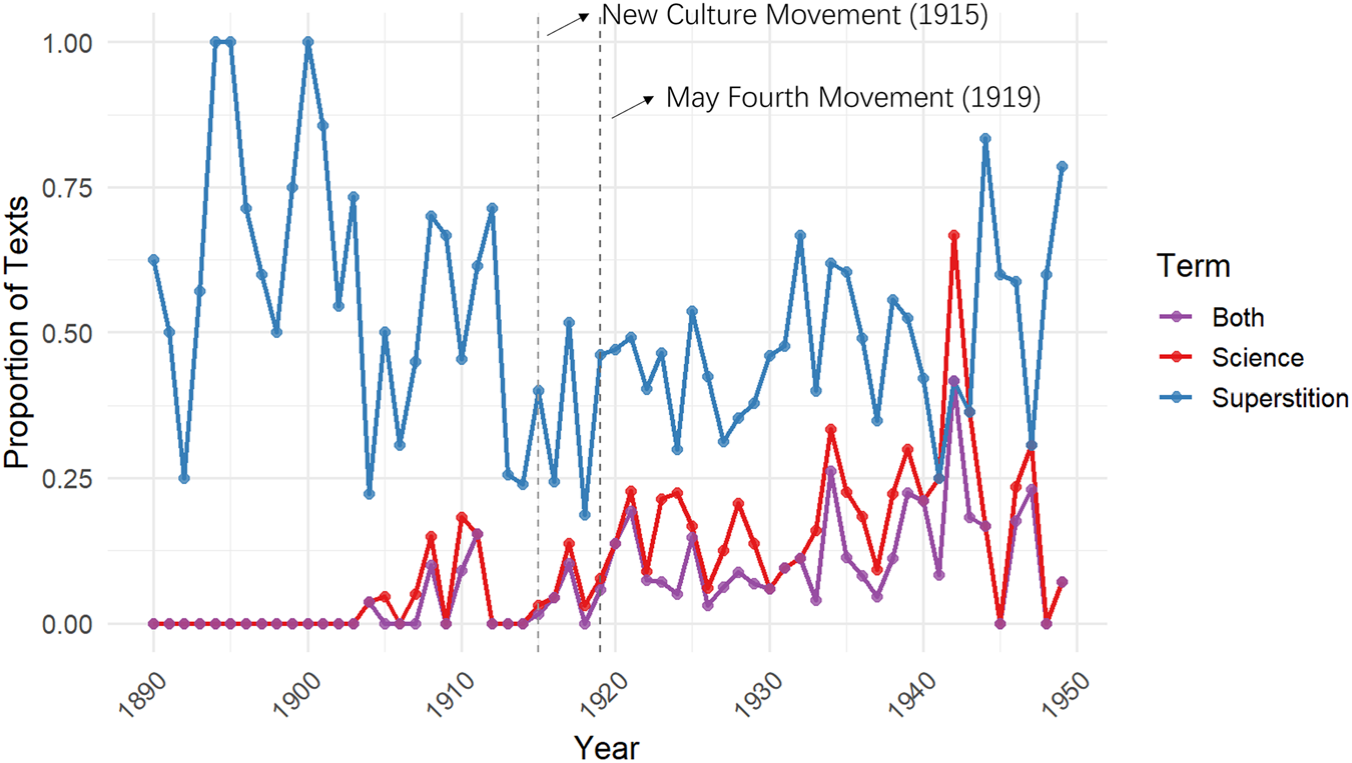
Proportion of texts mentioning “science,” “superstition” (mixin), and both terms over time (1890–1949).

Such accounts, grounded in specific events and eyewitness testimony, illustrate how proponents relied on vivid, verifiable‐seeming narratives to substantiate supernatural claims. By contrast, arguments rejecting ghosts and spirits often drew on abstract reasoning. Among the texts that argue against the theoretical plausibility of the existence of ghosts/spirits, about 25% explicitly mention the incompatibility between “science” (kexue 科學) and ghosts/spirits. Indeed, the newly imported concept “science” from the West and its contrast “superstition” (mixin 迷信, which literally translates as “excessive belief”) became a powerful rhetoric that depicted believers of ghosts/spirits as ignorant and unenlightened. Notably, over 60% of the texts that reference “science” also explicitly mention “superstition,” underscoring the polemical coupling of the two terms. Beginning in the early twentieth century—and especially after the New Culture Movement in 1915 and the May Fourth Movement in 1919—this rhetorical pairing became increasingly prominent. As shown in Fig. 5, the discourse of “science” was consistently positioned as the antithesis of “superstition.” As an example, a 1935 commentary in *Hankou Sino‐Western News* that laments the fact that lay people would seek ghosts/spirits for help in cases of illness concludes the following:

For several thousand years, our society has been dominated by theocratic superstition. Although science now flourishes and the government strictly prohibits superstitious practices involving ghosts and spirits, and although scholars have rigorously refuted tales of immortals and specters, the ignorant masses continue to revere and believe in them with undiminished fervor. Among these, the belief in seeking divine healing is especially deep‐rooted and difficult to reverse. Regardless of age or gender, whenever illness strikes, people instinctively turn to deities—inviting spirits, sending off ghosts, summoning immortals, or consulting oracles—treating these as their only options for recovery. Some even go so far as to entrust their precious lives to lifeless idols, entirely devoid of consciousness or rationality. The absurdity of such behavior is both laughable and pitiable.^12^

Such critiques reflect a broader intellectual current of the time: one that sought to discredit supernatural belief by aligning it with backwardness, and to promote scientific reasoning as the hallmark of a modern, rational society (Wu, 2022). These commentaries often positioned science not only as a methodological standard, but also as a moral imperative, casting belief in spirits as both an epistemic failure and a civic liability. Sometimes, authors went further by attributing such beliefs directly to a lack of modern education. They argued that limited access to schooling and widespread scientific illiteracy created fertile ground for ghost beliefs to persist:

In China, there are too few people who have received a complete education, and many lack basic scientific knowledge. When they encounter something they don't understand, they tend to attribute it to spirits or ghosts. Superstition about ghosts and spirits grows deeper by the day. Even those with only a shallow educational background are gradually assimilated into this belief system by society. During the late Qing period, temple demolitions and school‐building efforts were widespread across the country. But now, schools are gradually declining, while incense‐burning, Buddha‐worship, temple renovations, and statue‐making are increasing day by day. What's more, prominent individuals and high‐ranking officials are actively promoting such practices. People seem to be abandoning science in favor of studying “ghostology.” I find this trend deeply troubling.^13^

These arguments suggest that belief in supernatural agents was not only irrational but also socially contagious—spread through community norms, elite endorsement, and institutional neglect of secular education. As such, reform‐minded critics viewed the persistence of ghost belief as symptomatic of structural and cultural stagnation. At the same time, however, there were some scholars who attempted to reconcile the traditional concept of ghosts and spirits with a scientific worldview, sometimes by invoking the rationalizing of the traditional concept of “qi” (energy) as the basis of ghosts and spirits. Indeed, there were periodicals (e.g., *Spirit Studies Digest*) that were entirely dedicated to the (pseudo)scientific study of spiritualism.

In addition to science versus superstition contrast, more than half (53%) of arguments against the existence of ghosts/spirits employed psychological explanations to account for people's belief in them. These explanations typically reframed supernatural belief not as a reflection of metaphysical truth, but as a product of cognitive tendencies, emotional needs, or social pressures. In this view, belief in spirits is not only a truth‐claim to be debated, but also a phenomenon to be explained. Some authors, for example, attributed ghost belief to the mind's tendency toward imaginative elaboration: “Before the rise of modern science, no one could answer or explain how people come into being, how they die, or what happens after death. As a result, people imagined the existence of gods to govern life and death, and they imagined that humans have souls which, after death, become ghosts.”^14^ More commonly, belief in spirits was linked to fear, grief, or anxiety—emotional states in which individuals project meaning onto ambiguous events as a way of coping with uncertainty, misfortune, or loss. These explanations often drew on surprisingly modern psychological insights. One commentary from *Shenyang Higher Normal School Weekly* on spiritual healing practices observed:

“When it comes to witchcraft and healing, people in pain, whether physical or emotional, seek relief by any means, especially when conventional doctors are deemed unreliable. This desperation often leads to superstition, with people turning to witches believed to be endowed with spiritual powers, hoping for an end to their suffering…Before attempting to heal through spirits, the witch might demand silence about the process. The patient, under the psychological influence and hopeful words of the witch, may feel some relief due to a shift in mindset, even if there's no real effect. Should there be no improvement, it's attributed to breaking a taboo by speaking the truth. Furthermore, in families where some members oppose spirit possession rituals due to skepticism or fear of ridicule, there's often a reluctant agreement to remain silent about the lack of efficacy, driven by a desire to protect and support one another despite the pain.”^15^

This psychologizing discourse marked a departure from traditional ontological debates, instead treating belief itself as a phenomenon to be explained as part of a broader intellectual movement toward secularization and scientific rationalism. The presence of such psychological framing signals a growing effort, particularly among modernist and reformist writers, to naturalize and thereby delegitimize supernatural belief in addition to direct theological/ontological critique.

## 4 Discussion

This study offers the first large‐scale, systematic analysis of how ghosts and spirits were debated in public discourse during a period of profound intellectual transformation in China. By combining quantitative annotation of over 2000 historical texts with close reading and statistical modeling, we provide empirical support for the hypothesis that arguments against supernatural entities were primarily framed in theoretical rather than empirical terms.

The asymmetry between theoretical critiques and empirical defenses also suggests a divergence in cognitive and rhetorical strategies between skeptics and believers. Skeptics, often Western‐educated elites, leaned on abstract reasoning and scientific principles to challenge the plausibility of supernatural entities, reflecting their access to and alignment with global intellectual currents. In contrast, believers relied more on personal experiences and anecdotal evidence in voicing their support for the existence of ghosts/spirits, which were deeply rooted in cultural practices like ancestor worship and ritual divination.

Importantly, our findings should not be interpreted to mean that theoretical plausibility is irrelevant to the persistence of supernatural belief. On the contrary, belief in ghosts and spirits is often sustained within broader systems of thought—supernatural cosmologies that provide internal coherence and resilience against disconfirming evidence (Hong et al., 2024). It is precisely because these beliefs are anchored in such robust cultural and cognitive frameworks that they persist across time and context, even when challenged. We suggest that this embeddedness also explains why cultural elites felt the need to attack these beliefs at their conceptual core. For reformers and modernizers, discrediting ghosts and spirits was not merely about eliminating a set of outdated practices; it was about confronting the epistemological foundations of an entire worldview. The struggle over ghosts and spirits, in this sense, was emblematic of a broader cultural and ideological contest—between traditional metaphysics and the imported scientific rationalism, which leaves no room for supernatural entities.

Our findings underscore the central role that the discourse of science played in delegitimizing belief in ghosts and spirits. By aligning scientific reasoning with civic virtue and social progress, reformist intellectuals reframed supernatural belief as not merely false but socially harmful and intellectually regressive. The frequent pairing of “science” with its rhetorical opposite—“superstition” (迷信)—allowed critics to paint religious and folk practices as epistemically and morally backward. This binary was further sharpened by appeals to education: ignorance, rather than theological error, was often seen as the root cause of supernatural belief. In this sense, rejection of the supernatural became an index of cultural and cognitive advancement.

This framing can also be interpreted through the lens of prestige‐biased cultural transmission. As Henrich (2016) and others have argued, when a society perceives another group as more successful or authoritative, it tends to copy not just practical innovations but also ideological and institutional frameworks. In early twentieth‐century China, science was not only a method of inquiry but a prestigious Western institution. By aligning themselves with science and reason, Chinese elites signaled membership in a modern, progressive, and internationally respected epistemic community. Just as Western education, dress, and governance structures were adopted as symbols of advancement, the rejection of ghosts and spirits became a cultural marker of elite modernity.

That said, it would be a mistake to assume that science and supernatural beliefs were viewed as inherently incompatible. As Lamont (2013) notes, the late nineteenth and early twentieth centuries saw a flowering of scientific spiritualism in the West, with serious attempts to test spirit communication, telepathy, and mediumship using controlled experimental methods. A parallel interest emerged in China as well: some prominent Chinese intellectuals—such as Yan Fu, a famous educator and translator who introduced many Western ideas to China—engaged with spiritualist phenomena in a spirit of nondogmatic, empirical inquiry, seeking not to dismiss such experiences but to better understand them using the tools of modern investigation (Huang, 2007). These examples show that the perceived conflict between science and spirit belief was not absolute but shaped by broader ideological commitments and sometimes idiosyncratic personalities. While most reformers saw the two as fundamentally opposed, some sought a reconciliation that preserved the plausibility of ghost/spirit, despite the mockery and ridicule of such “ghostology.”

Equally noteworthy is the rise of psychological explanations, which accounted for more than half of the theoretical critiques in our corpus. In addition to directly refuting the existence of spirits, these arguments framed belief itself as a psychological phenomenon—an effect of fear, anxiety, habit, or suggestibility. The framing of belief in ghosts as a cognitive bias or emotional crutch represents a distinctly modern epistemic move, one that shifts the focus from *what is true* to *why people believe*. This psychologizing discourse parallels contemporary Western efforts to naturalize religion (Boyer, 2001; McCauley, 2000) but is distinct in its urgency, driven by China's need to assert sovereignty and cultural legitimacy in the face of colonial pressures (Goossaert, 2006).

Taken together, our findings shed new light on the intellectual and rhetorical strategies that shaped the secular transformation of Chinese society in the early twentieth century. By uncovering the argumentative asymmetries between skeptics and believers—and tracing how these stances were framed through scientific, psychological, and educational vocabularies—this study shows how supernatural belief became a central battleground in China's contested transition to modernity. Importantly, our work reveals a plurality of positions that went beyond simple belief or disbelief: alongside militant rejections and fervent affirmations, we find agnosticism, empirical caution, and attempts to reconcile spiritual traditions with scientific thought.

This is the new vista our study opens: it shifts the focus from *what* people believed to *how* they publicly argued for their positions, and in doing so, provides a methodological blueprint for re‐examining cultural change (Hong & Chen, 2024 ). The novel dataset, a richly annotated corpus of over 2000 historical texts, offers an unprecedented empirical foundation that allows us to move beyond the study of elite intellectuals and map the broader discursive patterns of an era. This resource opens new, specific possibilities for research. For instance, researchers can now quantitatively track the semantic shift in how “superstition” (迷信, *míxìn*) was framed—moving from a marker of personal ignorance in the 1910s to a threat to national progress and public health by the 1930s. Similarly, one could test whether the abstract, philosophical critiques of spiritualism common in elite journals were rhetorically ineffective against the vivid, first‐person testimonials that proliferated in popular newspapers, thereby explaining the resilience of belief outside intellectual circles in certain circumstances. Furthermore, the dataset allows for a fine‐grained analysis of “strategic appropriation,” examining precisely how believers selectively adopted the vocabulary of science—such as “energy,” “waves,” or “psychical research”—to lend modern legitimacy to traditional claims. By making the rhetorical strategies themselves the object of analysis, our work enables a new generation of research on religious cognition, secularization, and cultural change. Future work can extend this methodology to other non‐Western contexts, exploring whether the argumentative asymmetries we identified are distinctive to Republican China or reflect broader patterns in the diffusion of secular modernity. A key limitation, however, is that our study does not assess the downstream effectiveness of these discourses in altering public belief, an area awaiting future reception studies.

Looking forward, our findings invite dialogue with modern computational studies on persuasion. The argumentative asymmetry we uncovered (where skeptics favored abstract theory and believers leaned on personal experience) in some sense mirrors the findings of Na and DeDeo (2022), whose work on contemporary online debates shows that personal anecdotes are often more persuasive than abstract deduction. This suggests the rhetorical power of narrative over logic may be a persistent feature of human persuasion in certain contexts. This cross‐temporal resonance also highlights the value of cross‐cultural comparison. For instance, Scheffer, van de Leemput, Weinans, and Bollen (2021) identified a macro‐historical rise of rationalist language in Western‐language books since 1850. A compelling avenue for future research would be to juxtapose the specific rhetorical dynamics of Republican China with such large‐scale linguistic trends observed elsewhere. This comparative work would be invaluable for building more generalizable theories and constructing nuanced hypotheses about the interplay between language, culture, and societal change.

## Funding

The work was supported by The John Templeton Foundation (JTF grant ID# 61928). Ze Hong was additionally supported by SRG2023‐00041‐FSS from the University of Macau.

## Supporting information



Supplementary Information

## Data Availability

All data and analysis files can be found at https://osf.io/vqxe7/?view_only=41045621e3fe4b919cea75203eb93a38

## Notes

1 Original text: 悉天地間有主宰焉。雖大至無外。小至微塵。莫不由其意匠之所告也。此一神教之所由生也。天地間雖一草一木。莫不有其專屬之神。善惡因果之報。不獲罪於鬼神。此多神教之所由設也。生死周循。善惡爲報。即小至疾病天壽。福禍災殃。莫不有其來因去果。而歸其終點於天堂地獄之分。使人類視聽言動。一循天理之正。雖杳冥不可彷彿。而亦若有戒慎恐懼之心。勿敢爲非作惡。此宗教之所由用也。是故有宗教而天地間一切疑難勿可解決之問題。Source: 蔡孑民在信教自由會之演說, 東方雜志Dongfang zazhi, Mar. 15, 1917, V.14, Num. 3.

2 Original text: 以宗教養成之道德, 系從畏敬恐懼而來, 不過如佞佛之老嫗, 奏獎之善士, 能爲鄉黨自好之士, 已爲不可多得之人。以教育養成之道德, 必有剛毅活潑之氣, 方將衝破一切之障礙, 革新現在之社會. Source: 論宗教妨害社會之進步, 申報Shen Pao, March 1, 1908.

3 Original text: 梅雨連綿, 氣候不正, 民間疎於調攝, 即爲造化小兒所侮, 西鄉一帶, 病者尤多。值此農事方興之際, 小民皆竊竊憂之, 議舁瘟神以驅疫癘。然不講衛生之法, 而惟從事於鬼神, 抑何愚也！Source: 禹穴探奇, 申報Shen Pao, July 5, 1903.

4 See Supporting Information for original text. Source: Hunan Provincial Government Gazette湖南省政府公报, 1934, issue 188, pp. 77−81.

5 Original text:鬼神之有無目下世界各國都在研究中我人不可必其爲有亦不可必其爲無金我國素以迷信鬼神著稱於世界然者古人神道設教原是用以患心誡末俗輔助法律之不逮者也後人不察愛本加厲遂貽莫大譏羞於世界此豈初精所及哉金自科學昌明鬼神之說漸廢年來靈魂學盛行鬼神之說又興於是鬼神有無之問題乃大費研究我人惟有稍安毋躁以待彼研究者之最後報告耳. Source: 新世界 New World, March 5, 1919.

6 See https://osf.io/dg629/overview?view_only=9d8b24a38bf84305913d67bb62aa3266

7 Original text: 易曰精气为物游魂变, 是故知鬼神之情状, 夫天地之间, 孰非物也？孰非魂也？孰非变也？Source: 字林滬報 Shanghai North China Herald, 1882, September 18.

8 Original text: 劉白, 南邑人, 性狂放不羈, 生平尤不信鬼神, 興談之者多被斥……某日夜歸經村, 聞後有追逐聲, 數顧無見, 知有異, 遂呼曰:「汝是好男兒, 可蜒身而出, 與爾公決勝負！」……空中頓現一掌拍劉面, 痛不可忍……翌日視面, 紅點點如壽陽, 數年始退……自此不敢再示鬼神矣. Source: 神州日报Shenzhou Daily, 1918, August 28.

9 Original text: 余歎夫吾國人民之信鬼并笑夫吾國愚民之懼鬼而不自知巳之心理作用神經作用以致迷信相延歷數千年而不破. Source: 神州日报 Shenzhou Daily, 1917, April 26.

10 Original text: 雖經我和父親竭力勸導却一依舊不能够勸醒地一眞神靈遇到意外的大禍了一由是地老人家對於祝神睹一堪浩嘆祭鬼頗爲忙碌最可嘆的確爲每月的拜佛但是心妯雖然如此勤敵誠結果却毫無得益上反消耗了許多無謂. Source: 妇女杂志(上海) Women's Magazine (Shanghai), 1925, Vol. 11, issue 11, pg. 1799−1800.

11 Original text: 木埠法界某香粉店主胡知去年暴病身亡. 遺有一妻二子長子十六次子五歲前日長子亦病死其母舐犢情切痛不欲生胡某忽附靈於次子之 身而言 日 母遇哀傷子 命也.長子在日作惡多端任性妄為今已露冥司罰入餓魂渤某街某姓家矣。 轉生江北其妻丕信親往訪抄其家果 於是日舉一男. Source: 时事新报(上海) The China Times (Shanghai), April 4, 1918.

12 Original text: 吾國社會。受支配於神權迷信之下者數千年。今雖科學明昌勺政府對鬼神迷门之事嚴加取締學者於神仙鬼怪之說力闢其誹而一般愚民仍崇拜之信仰之不稍衰尤以求神治病之迷信為最積重协返。無侖男敦老少〇偶燕疾病)辙皂皇然求神送鬼。請仙扶乩若舍別無措死回牛之術也者不惜舉寶貴生命付托於毫無知覺與理生之偶象其註味荒謬可笑亦可憐. Source: 汉口中西报 Hankou Sino‐Western News, March 6, 1935.

13 Original text: 中國因爲受過完全敎育的人太少缺少科學常識遇到不明白的事就往神鬼上附㑹迷信鬼神的程度一天比一天深把受過敎育根底淺的也就被社㑹同化了在前清時代各地毀廟辦學的事情很多到了現在學校漸減而燒香佛修廟塑像的事情日益增加又加達人顯宦之提倡人皆將棄科學而研究鬼學了我對這種事情很發愁. Source: 沈阳高等师范学校周刊 Shenyang Higher Normal School Weekly, 1921, volume 38, pp. 18−21.

14 Original text: 在科學沒有倡明之先了無人能囘答解釋人是怎樣㑹生了怎樣㑹死的死後又怎樣了因此就想像出神來支配人的生死了想像出人有靈魂了死後就變成鬼. Source: 敬中学生期刊Jingzhong Students’ Periodical, 1936, Issue 2, pp. 66−70.

15 每有好奇的心故意把夢先後倒置合事實湊合揚言於人說鬼神有靈來托夢聽的就信爲眞鬼神的靈驗豈不可笑嗎巫之治病凡人有病時身體精神之痛苦無不求解脫的因爲醫生都不可靠心過於切遂就迷信起來了往往認巫有鬼神之靈希望他給解脫痛苦但平常人也不信仰病人願意雖家庭有一部分人不信也就俯從病人的意思鬼神治病之前巫就要求啦不准說破話當時病人受一種心理作用精神的變動隨巫人的言語爲轉移也就覺著病體稍輕假使毫不見效也就因說實話犯了忌諱再有家裡有一部分反對跳神的跳神無效驗因爲怕人譏笑合護短的心理鄕忍痛一時不說. Source: 沈阳高等师范学校周刊 Shenyang Higher Normal School Weekly, 1921, volume 38, pp. 18−21.
