# Supplementary material for: The Rhetoric of Disenchantment: Ghost Belief and Secular Critique in Early Twentieth‐Century China
Source: Cogn Sci. 2026 Jan 19;50(1):e70158. doi: 10.1111/cogs.70158 (PMC12815379; doi:10.1111/cogs.70158)
Supplement: Supplementary file 1 — Supplementary Information [file COGS-50-e70158-s001.docx]

Supplemental Information for
“The Rhetoric of Disenchantment: Ghost Belief and Secular Critique in Early Twentieth-Century China”

1. Search strategy and exclusion criteria

As mentioned in the main text, all of our texts were obtained from two large, online databases of late Qing and early Republic Chinese periodicals. In the online version of Shanghai Library (<https://www.cnbksy.com/>), we used the keyword “鬼神” in the “Advanced Search” type and specified the search mode as “Precise”, and excluded a common metaphorical use of the “鬼神” in “泣鬼神” (literally, to move ghosts and spirits to tears) by using the “Not” function provided by the database. In the Hantang database, we employed the same search strategy using the “Exact” search mode and the “Not in result” function to excluding results containing “泣鬼神”. Further exclusion of irrelevant texts was based on human coders’ subjective judgment.

2. Annotation protocol and inter-rater reliability check

We initially recruited three human coders, each responsible for annotating one-third of the extracted texts (compiled in the dataset result_all_coder). The full annotation instructions provided to the human coders are available in the Supplemental Information (SI_codebook). In addition, we used ChatGPT-4.0 to annotate the same texts using prompts closely aligned with the human coding instructions. While a broad set of variables was initially coded, only those with acceptable inter-rater reliability are reported in the main text. Variables with low reliability were excluded from subsequent analyses. Notably, we found that one coder's annotations consistently exhibited questionable quality. As a corrective measure, we replaced that coder's data with the corresponding annotations generated by ChatGPT (compiled in the dataset result_all_coder_updated).

Supplementary Information Table 1 presents inter-rater reliability statistics (Fleiss’ Kappa) for all coded variables. Among these, we had originally distinguished two types of empirical justifications regarding belief in ghosts and spirits. *Empirical_specific*—used in the main text—refers to arguments grounded in individualized experiences cited as evidence for or against supernatural entities. *Empirical_general* refers to broader claims based on presumed general empirical patterns or inductive reasoning. Upon evaluating inter-rater reliability, we found that *Empirical_general* had particularly low agreement and therefore excluded it from further analysis.

| Variable | Fleiss’ Kappa, HJN, ZX, ZY | Fleiss’ Kappa, LLM, ZX, ZY | Fleiss’ Kappa, ZX, ZY |
| --- | --- | --- | --- |
| If_relevant | 0.103 | 0.35 | 0.232 |
| existence | 0.598 | 0.592 | 0.81 |
| utility | 0.0346 | 0.374 | 0.213 |
| morality | 0.0141 | 0.13 | 0.00165 |
| theoretical | 0.194 | 0.336 | 0.483 |
| Empirical_general | -0.0347 | 0.11 | -0.0667 |
| Empirical_specific | 0.13 | 0.312 | 0.462 |
| psychological | 0.292 | 0.396 | 0.438 |
| qi | 0.156 | 0.458 | 0.262 |
| mixin | 0.736 | 0.585 | 0.686 |

SI Table 1. Inter-rater reliability test statistics for different raters (including ChatGPT, denoted as "LLM").

3. Original text for footnote 4:

迷信心理之謬誤及鬼神之真義何主席十月十五日在擯大紀念週中講演近來報紙上〇常常登載迷信受害的新聞〇在民權昌明的今日口而少數人仍然脫不了神權時代意味〇智識簡單口可笑更復可憐〇現在想合這一般人口絕對不信鬼神口事實上頗不容易〇但是信有信的方法口不能入迷〇今天且就絕對迷信鬼神口與絕對不信鬼神的兩方面〇略說其故口以袪羣迷〇凡迷信家才力所不能解决的問題口都要去問卦求籛〇取决於神口好像他們所說的菩薩靈官等名號〇確實的有一位眞神口惱坐上面影誦通廣大口無所不能〇並且一無所事口專管人們的禍福休咎一般〇究竟有沒有靈驗口恐怕迷信者所說的靈驗事實〇多係聽自傳說口以極平常的道理論〇兒女有求於父母〇朋友有求於朋友口尚且不能有求必應〇鬼神與你們有什麽關係〇你有求於鬼神〇鬼神何求於你口以素不相干的人〇因爲你磕丁幾次頭口上丁幾炷香口說丁幾句諂媚的話口就答應你的要求〇是鬼神也行賄賂同人世一樣了〇此等鬼神〇便是卑污苟賤斗筲無賴之徒根本上就喪失爲鬼神的資格如何能降福與你論來鬼神之有無〇乃另一問題〇從有的方面說口人有人界口鬼有鬼界口神有神界其對於人的關係也只如商人對於工人工人對於農人農人對於士子〇彼此各有各的環境〇不相干涉〇商人旣沒有管農人的權力〇農人也沒有管工人的必要〇普通一般鬼神爲什麽要管人的事呢職掌人事的鬼神正直無私〇决不隨便許你請求〇古人常說一聰明正直之謂神一神旣然聰明〇就不㑹聽人的鬼話〇既然正直就不㑹應人無理之求〇可知妄求是沒有用的〇况且書經上說一作善降之百祥作不善降之百殃一〇有自然而然之勢〇人們作善口鬼神不得不降之以福〇人們作惡口鬼神不得不降之以禍禍福惟人自召〇鬼神並不能操其權柄所以孔子說〇一獲罪於天無所禱也一由此處體〇人們求鬼神〇不如求自已〇凡理屈而氣不壯的人心所禱鬼神〇就等於自投羅網不如故過還善或可解除罪惡〇此外又有一般人口絕對不信有鬼神〇以爲現代科學昌明〇凡實在的事口都可用科學方法口實驗出來〇鬼神旣無形可見〇無聖可聞又不能用科興證明所以鬼神是絕對沒有的〇此說與上說完全居於相反地位〇我們也不能加以贊成何以呢〇宇宙閒的事理無窮口而人的知識有限口其事理之所有口而爲人類智識之所未及者〇不知道有若干萬萬口自已的智識不及口付之缺疑則可〇若以自已所不見不聞的事口就武斷說爲未有〇試問從前誰不說水裏空氣裏口以及種種傳染病裏裏面什麽都沒有〇爲什麽自法人也斯德發見微生物口後口人人又都說水裏空氣裏有微生物口傳染病裏有毒菌呢〇由此推說〇凡科學不能證明的事〇只是科學的程度未到不能說鬼神確實沒有〇硬說沒有的人口不惟藐視玄學理解不全〇其對於科學至多也只是一知半解〇沒有高深的認識

4. Temporal trend of the relative frequency of ghost/spirit mentions

To control for the increasing volume of newspapers and periodicals over time, we normalized the annual count of articles mentioning "ghosts" or "spirits." An ideal normalization would use the total number of articles published per year as a denominator; however, this information could not be directly extracted from the databases. To create a proxy for this total, we focused on the Shanghai Library database and performed a keyword search for the space character (" ") within the title field, collecting the resulting article counts for each year. We then calculated the relative frequency of ghost and spirit mentions against this proxy value (SI Figure 1). Two data points from early years (1868 and 1878, with one and three mentions respectively) were excluded from this analysis. Due to the very low total document count in this early period, these points produced unstable frequency estimates that would have distorted the visualization of the overall temporal trend. After normalization, the analysis confirms the same qualitative pattern: mentions of ghosts and spirits remain concentrated in the period from the 1910s to the 1930s.


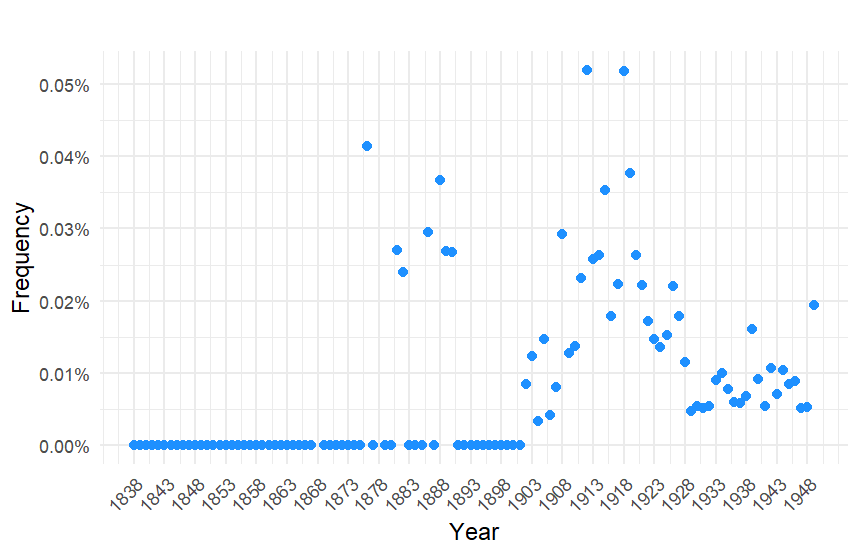


Figure SI 1.Normalized temporal trend of ghost and spirit mentions. This figure displays the relative frequency of articles mentioning "ghosts" or "spirits," normalized against a proxy for total annual publications to control for publishing volume.
